# Diabetic Kidney Disease: Pathophysiology and Therapeutic Targets

**DOI:** 10.1155/2015/697010

**Published:** 2015-04-30

**Authors:** Stephanie Toth-Manikowski, Mohamed G. Atta

**Affiliations:** Division of Nephrology, Johns Hopkins University, 1830 E. Monument Street, Suite 416, Baltimore, MD 21287, USA

## Abstract

Diabetes is a worldwide epidemic that has led to a rise in diabetic kidney disease (DKD). Over the past two decades, there has been significant clarification of the various pathways implicated in the pathogenesis of DKD. Nonetheless, very little has changed in the way clinicians manage patients with this disorder. Indeed, treatment is primarily centered on controlling hyperglycemia and hypertension and inhibiting the renin-angiotensin system. The purpose of this review is to describe the current understanding of how the hemodynamic, metabolic, inflammatory, and alternative pathways are all entangled in pathogenesis of DKD and detail the various therapeutic targets that may one day play a role in quelling this epidemic.

## 1. Introduction

Diabetes has long been a growing epidemic in the United States (US) and around the world. In 2011, there were 20.8 million people aged 18 years and older who carried a diagnosis of diabetes in the US alone [1]. The number of adults aged 18–79 in the US that were newly diagnosed with diabetes has more than tripled from 493,000 in 1980 to over 1.5 million in 2011 [2]. The increased prevalence of diabetes has also led to an increase in the number of macro- and microvascular complications of diabetes such as coronary heart disease, stroke, visual impairment, diabetic kidney disease (DKD), and end stage renal disease (ESRD). Additionally, diabetes remains the most common reason for progressing to end stage renal disease in the US and in many parts of the world [3–5]. The number of people initiating treatment for ESRD related to diabetes was 48,374 people in 2008, more than 18-fold what it was in 1980 [6]. DKD was previously known as diabetic nephropathy and is defined as diabetes with albuminuria (ratio of urine albumin to creatinine ≥ 30 mg/g), impaired glomerular filtration rate (<60 mL/min/1.73 m^2^), or both and is the single strongest predictor of mortality in patients with diabetes [7]. Today, DKD encompasses not only diabetic nephropathy but also atheroembolic disease, ischemic nephropathy, and interstitial fibrosis that occurs as a direct result of diabetes.

Glycemic control and RAAS inhibition have long been mainstays of therapy in patients with DKD. Multiple large trials have demonstrated that improved glycemic control in patients with type 1 and 2 diabetes reduced microalbuminuria [8, 9], macroalbuminuria [8, 9], and progression to DKD and ESRD [9, 10]. RAAS inhibition with angiotensin converting enzyme inhibitors (ACEIs) and angiotensin receptor blockers (ARBs) also reduces microalbuminuria and progression to DKD and ESRD [11–14]. Their benefit is largely attributed to reduced vasoconstriction of the efferent arteriole which consequently reduces hyperfiltration. Although regression of microalbuminuria has been documented in patients with both type 1 and type 2 diabetes, it has been demonstrated to be irreversible in African Americans [15]. Finally, ACEIs are currently recommended for primary prevention in patients with diabetes even in those without evidence of chronic kidney disease (CKD) [16, 17]. Beyond these widely known recommendations, clinicians have little else to offer patients with DKD.

## 2. Histopathology of DKD

**Figure 1:**
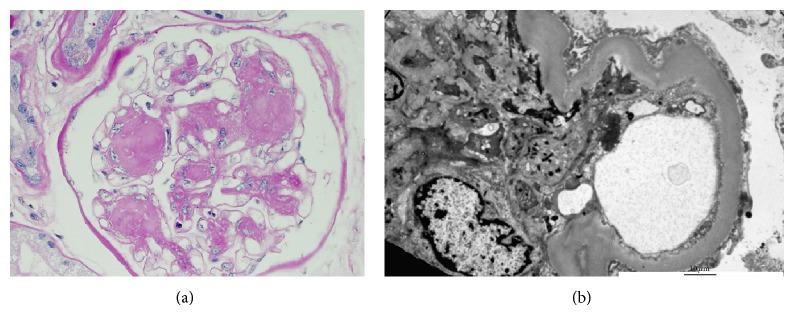
(a) Light microscopy with hematoxylin-eosin staining reveals extensive mesangial expansion without marked increase in cellularity. A Kimmelstiel-Wilson (KW) lesion is shown here and refers to the nodular glomerulosclerosis that can be seen in late disease but is not as common as diffuse diabetic glomerulosclerosis. KW lesions are usually spherical and eosinophilic and have a central hypocellular or acellular area. Mesangial expansion and KW lesions are both due to increased extracellular matrix production. (b) Electron microscopy reveals a thickened basement membrane and podocyte foot process effacement.

The histopathologic changes of DKD have been well documented previously and will not be described in detail here. Mesangial expansion caused by increased matrix secretion and cell enlargement is the first change seen on light microscopy, whereas electron microscopy demonstrates a thickened basement membrane and podocyte effacement (Figures 1(a) and 1(b)) [7]. In the vessels, intimal hyaline thickening is present initially and later progresses to arterial hyalinosis of the afferent and efferent arterioles which later leads to glomerular hyperfiltration [18, 19]. Diffuse diabetic glomerulosclerosis and Kimmelstiel-Wilson nodules (nodular glomerulosclerosis) are seen only later in the disease, although the latter is not always seen on biopsy as is classically taught [20]. Ultrastructurally, podocytes suffer hypertrophy and then foot process effacement which leads to functional changes such as increased albumin excretion [7, 18]. It should be noted that, in patients with type 2 diabetes, GFR loss can occur independently of albuminuria [19, 21, 22] and it has been demonstrated that microalbuminuria is observed in only 45% of this population [23]. The histopathologic change of DKD has been attributed to diabetic macroangiopathy as opposed solely to microangiopathy and has also been attributed to aging, atherosclerosis, hypertension, and episodes of acute kidney injury [19, 22, 24].

## 3. Pathways of DKD

Previously, the above histopathologic changes were attributed primarily to metabolic and hemodynamic derangements seen in diabetes, the latter referring to the hyperfiltration which occurs as a result of efferent arteriolar vasoconstriction due to an activated renin-angiotensin-aldosterone system (RAAS). However, it has become increasingly evident over the years that hyperglycemia in and of itself is not the sole cause of DKD, although inarguably, it plays a major role. Several pathophysiologic pathways are involved in the development of DKD, and this review will attempt to elucidate those pathways and hopefully shed some light on therapeutic options that may one day play a role in quelling the epidemic of DKD and suppressing progression to ESRD.

## 4. Hemodynamic Pathways of DKD

Activation of the RAS leads to increased angiotensin II levels which subsequently cause efferent arteriolar vasoconstriction. Elevated levels of angiotensin II are associated with increased albuminuria and nephropathy in both humans and mice [18, 25, 26]. ACEIs and ARBs have a long track record in reducing the doubling rate of creatinine, albuminuria, and progression to nephropathy, ESRD, and death [11, 13, 14, 27]. Another potent vasoconstrictor of the efferent arteriole is endothelin-1 (ET-1). ET-1 has various physiologic functions in the kidney that mimic RAS including mediating vasoconstriction and hence playing a role in hypertension, endothelial dysfunction, inflammation, and fibrosis [28]. Additionally, increased ET-1 expression activates a signaling cascade which leads to mesangial cell hypertrophy and proliferation as well as extracellular matrix (ECM) production. It is also thought to activate receptors that directly increase glomerular permeability, hence leading to worsening albuminuria and progression of DKD [28].

## 5. Metabolic Pathways of DKD

**Figure 2:**
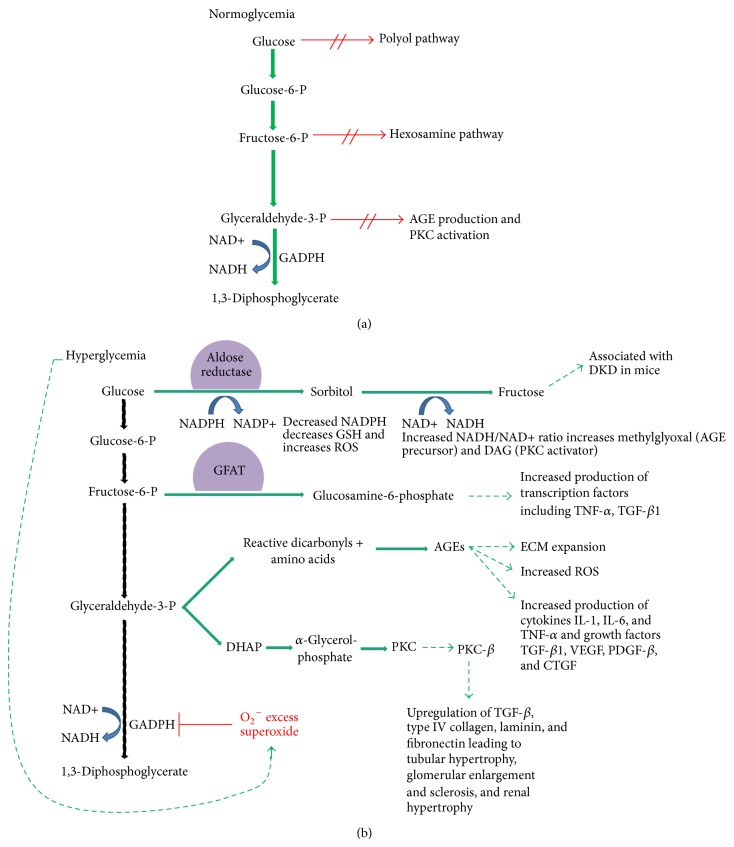
(a) Glycolysis is the biochemical pathway in which glucose is broken down by cells to make energy. In a normoglycemic environment, that is, in patients without diabetes, glycolysis proceeds down its well described path without shunting into the polyol pathway, hexosamine pathway, or pathways that would lead to AGE production or PKC activation. (b) In a hyperglycemic environment, as would be seen in patients with either type 1 or type 2 diabetes, high glucose conditions lead to activation of excess superoxide which then inhibits the enzyme GADPH. This prevents glycolysis from proceeding down its natural course and creates a backlog of glycolysis precursors. Increased levels of glucose upregulate the polyol pathway whereas increased levels of fructose-6-phosphate upregulate the hexosamine pathway. Increased levels of glyceraldehyde-3-phosphate upregulate both AGE precursors and DAG, the latter being a cofactor for PKC activation.

This pathway was first detailed by Brownlee in Nature in 2001 [29]. He helped clarify that hyperglycemia leads to increased glycolysis which then upregulates four distinct entities: the polyol pathway, hexosamine pathway, production of advanced glycation end products (AGEs), and activation of protein kinase C (PKC). Before going into the details of each of the above pathways, a review of glycolysis is worthwhile. Glycolysis is the biochemical pathway in which glucose is broken down by cells to make energy. Intracellular glucose is first broken down into glucose-6-phosphate and then fructose-6-phosphate. One step later glyceraldehyde-3-phosphate becomes 1,3-diphosphoglycerate with the help of glyceraldehyde-3-phosphate dehydrogenase (GADPH) (Figure 2(a)). This is important because GADPH is inhibited by excess superoxide produced by the electron-transport chain which occurs in the setting of hyperglycemia [29–31]. Inhibition of GADPH prevents glycolysis from taking place and causes an upregulation of upstream components of glycolysis, specifically glucose, glucose-6-phosphate, and fructose-6-phosphate (Figure 2(b), hyperglycemia).

### 5.1. The Polyol Pathway

The polyol pathway is upregulated as a result of excess of hyperglycemia. Glucose is first converted to sorbitol via the NADPH-dependent enzyme, aldose reductase; sorbitol is then converted to fructose using NAD+ as a cofactor [29] (Figure 2(b)). The reduction of glucose to sorbitol results in decreased intracellular NADPH levels, a cofactor involved in regenerating the antioxidant, reduced glutathione (GSH). Decreased levels of GSH are thought to contribute to increased intracellular oxidative stress which in turn causes increased cell stress and apoptosis [32]. Additionally, the oxidation of sorbitol to fructose results in an increased intracellular NADH :  NAD+ ratio which also inhibits GADPH activity, thus propagating the inhibition of glycolysis. The increased NADH : NAD+ ratio also increases formation of methylglyoxal and diacylglycerol, precursors of the AGE and PKC pathways which are discussed below [29]. Finally, the end product of the polyol pathway, fructose, has also recently emerged as a potential nephrotoxin. In a diabetic murine model, endogenous production of fructose through the polyol pathway led to increased proteinuria, reduced GFR, and increased glomerular and proximal tubular injury when compared to mice with lower levels of endogenous fructose. Additionally, these mice also expressed more superoxide levels and the inflammatory cytokine NF-*κ*B [32, 33], the importance of which will also be discussed below.

### 5.2. The Hexosamine Pathway

The hexosamine pathway stems from the third step of glycolysis, fructose-6-phosphate, which is converted to glucosamine-6-phosphate by the enzyme glutamine: fructose-6-phosphate amidotransferase (GFAT) (Figure 2(b)). Glucosamine-6-phosphate is then used as a substrate to increase transcription of inflammatory cytokines tumor necrosis factor-*α* (TNF-*α*) and transforming growth factor-*β*1 (TGF-*β*1) [29]. Increased TGF-*β*1 levels are known to promote renal cell hypertrophy and increase mesangial matrix components, two pathologic hallmarks of DKD [34, 35], whereas TNF-*α* is an inflammatory cytokine discussed in greater detail below.

### 5.3. Advanced Glycation End Products

Advanced glycation end products (AGEs) are the result of irreversible glycation of proteins that occurs in the presence of intracellular hyperglycemia [18, 29, 36]. Three pathways are primarily responsible for the production of AGE precursors: oxidation of glucose to make glyoxal, degradation of Amadori products, and aberrant glycolysis which shunts glyceraldehyde-3-phosphate into forming methylglyoxal (Figure 2(b)) [37]. Once formed, AGEs damage cells by modifying or impairing the function of both intracellular and extracellular proteins [36]. For example, AGE modifies both laminin and type IV collagen and was shown to increase the permeability of the glomerular basement membrane (GBM) [38–41]. Additionally increased concentrations of AGE are known to dose-dependently increase expression of fibronectin and collagen types I and IV which are thought to lead to increased density and expansion of the extracellular matrix in the kidney [38, 42–46]. AGEs themselves can bind various proinflammatory receptors which then activate downstream production cytokines such as IL-1, IL-6, and TNF-*α*, growth factors such a TGF-B1, vascular endothelial growth factor (VEGF), platelet-derived growth factor subunit B (PDGF-B), connective tissue growth factor (CTGF), and increased generation of reactive oxygen species (ROS) [19, 38, 47, 48]. VEGF is necessary for survival of endothelial cells, podocytes, and mesangial cells whereas CTGF is a profibrotic agent; both have been implicated in diabetic nephropathy [38, 49, 50].

### 5.4. The PKC Pathway

The PKC pathway, like the AGE pathway, stems from the fourth step in glycolysis (Figure 2(b)). Hyperglycemia drives the conversion of glyceraldehyde-3-phosphate into dihydroxyacetone phosphate (DHAP) and ultimately diacylglycerol (DAG) which is a cofactor for PKC activation [51]. In the presence of hyperglycemia, DAG is chronically upregulated and contributes to sustained PKC activation [52]. PKC is thought to contribute to DKD in various ways. It increases activity levels of prostaglandin E_2_ and nitric oxide [53–55] leading to vasodilation of the afferent arteriole and augmentation of angiotensin II's actions on the efferent arteriole [56, 57]; these actions collectively contribute to glomerular hyperfiltration [51]. In the later stages of diabetic nephropathy, there is a state of progressive deficiency in nitric oxide which has been associated with severe proteinuria, declining renal function, and hypertension [58, 59]. PKC also mediates VEGF which, as noted above, is linked to abnormal intrarenal blood flow and capillary permeability and is thought to play a role in the development of microalbuminuria [51, 60]. PKC activation also increases CTGF and TGF-*β* levels as well as production of fibronectin and type IV collagen and contributes to GBM thickening and ECM accumulation [51].

## 6. Inflammatory Pathways of DKD

The inflammatory pathway supports the idea that DKD is not solely a result of uncontrolled hemodynamics and hyperglycemia but is also a consequence of a chronically activated innate immune system and a low-grade inflammatory state in patients with diabetes [61, 62]. Inflammatory-mediated renal injury was reviewed recently and is summarized here [61].

NF-*κ*B is a transcription factor that regulates the expression of multiple genes related to inflammation, immunity, apoptosis, and chemoattractant protein-1, amongst others [63, 64], and localizes to glomerular, interstitial, and tubular epithelial cells in the human kidney. Hyperglycemic conditions are known to increase expression of NF-*κ*B [65]. In DKD [63, 66], NF-*κ*B activation correlates with proteinuria and interstitial cell infiltration [63, 64, 66]. Proteinuria is known to further stimulate NF-*κ*B and contributes to persistent proteinuria in a cyclic fashion [64].

The Janus kinase/signal transducers and activators of transcription (JAK/STAT) signaling pathway is a way for chemical signals outside of a cell to be relayed to gene promoters at the DNA level. JAK2 is present in renal and vascular tissue [67]. It is activated by ROS caused by hyperglycemic states and is associated with hypertrophy of mesangial cells [61]. Berthier et al. demonstrated that JAK2 mRNA levels inversely correlated with estimated glomerular filtration rate (eGFR) in patients with diabetic nephropathy [68].

Inflammatory cytokines such as TNF-*α* and interleukins 1, 6, and 18 (IL-1, IL-6, and IL-18, resp.) are expressed in greater proportions in the kidneys of diabetic models when compared to nondiabetic controls [69, 70]. In diabetic rat models, increased expression of TNF-*α* and IL-6 was also associated with increased kidney weight and urine albumin excretion [69]. In patients with DKD, serum IL-18 and TNF-*α* levels were higher in patients with diabetes than nondiabetic controls. IL-18 and TNF-*α* levels also correlated positively with the degree of albuminuria in the patients with diabetes [71, 72]. At the cellular level, these cytokines are thought to increase vascular endothelial cell permeability, contribute to glomerular hypercellularity and GBM thickening, induce apoptosis of endothelial cells, and can be directly toxic to renal cells [73–81].

## 7. Alternative Pathways of DKD

Autophagy is a highly conserved protective mechanism that allows cells and organisms to maintain homeostasis during periods of cell starvation or oxidative stress [82, 83]. It involves intracellular degradation of cytotoxic proteins and organelles by lysosomes whenever a cell is experiencing stress [83, 84]. Decreased autophagic activity has been demonstrated in both obesity and diabetes [85–87] suggesting that autophagy is hampered in the setting of hypernutrition [88]. Podocytes are known to have a high basal level of autophagy [87].* In vitro* studies of podocytes exposed to high glucose conditions demonstrated defective autophagy which resulted in podocyte injury [87]. On renal biopsy of obese patients, autophagic activity was decreased in proximal tubular epithelial cells when compared to nonobese patients suggesting that obese patients with diabetes may be prone to renal injury due to suppressed autophagy [88]. Dietary restriction in rats was shown to improve urinary albumin excretion and creatinine clearance and increase levels of Sirt1, a positive regulator of autophagy [89].

Another conserved evolutionary mechanism is linked to the sodium-glucose transporter 2 (SLGT2) in the proximal tubule. SLGT2 is a low-affinity and high-capacity transporter and is responsible for >90% of glucose reabsorption in the proximal tubule [37, 90, 91]. Animals with a genetic deficiency of SLGT2 lose approximately 60% of their filtered glucose into the urine [90]. In settings of hyperglycemia, there is upregulation of SLGT2 expression which is believed to be of evolutionary benefit as it allows for glucose reabsorption and hence energy conservation for both the body and brain [90, 92]. Unfortunately, in settings of hyperglycemia due to diabetes, this mechanism is counterproductive and further contributes to a hyperglycemic state.

## 8. Therapeutic Agents Targeting the Hemodynamic Pathway

ET-1 antagonists first showed promise in diabetic rat models when they were compared to ACEI and had significantly decreased renal glomerular diameter and deposition of eosinophilic material within glomeruli [93]. In another experimental model, the ET-1 antagonist avosentan demonstrated attenuated mesangial and glomerular matrix protein accumulation as well as normalization in creatinine clearance; these findings were comparable or superior to mice that had been randomized to ACEI [94]. The ASCEND trial was a multinational, double-blind, placebo-controlled trial which randomized patients with type 2 diabetes with overt nephropathy to avosentan or placebo in addition to continued RAS-inhibition. Although the trial was stopped prematurely due to an excess of cardiovascular events in the intervention group, there was a dose-dependent reduction in albuminuria in the avosentan group [95] when compared to the placebo arm. A post hoc analysis of the ASCEND trial found that the increased events of congestive heart failure (CHF) were preceded by increases in body weight and that future trials with ET-1 receptor antagonists would benefit from close monitoring of body weight to sooner identify any potential CHF development [96]. In a more recent study, data from two-phase 2b, randomized, double-blind, placebo-controlled trials in patients with type 2 diabetes with overt nephropathy were pooled to compare concomitant atrasentan and RAS-inhibitor use with a placebo group. Compared to placebo, the atrasentan/RAS inhibitor group had a dose-dependent improvement in albuminuria. While there was also a significant increase in body weight, the rates of cardiovascular events did not differ between the groups [97]. The SONAR trial is currently undergoing large-scale recruitment and will evaluate the effect of concomitant administration of atrasentan and RAS inhibitor on firm clinical endpoints such as the first occurrence to a composite renal endpoint, doubling of serum creatinine, or the onset of ESRD [98].

## 9. Therapeutic Agents Targeting the Metabolic Pathway

Aldose reductase inhibitors prevent the conversion of glucose to sorbitol by inhibiting the enzyme aldose reductase. Epalrestat was shown to prevent mesangial expansion and improve urine albumin excretion in diabetic rats [99, 100]. In patients with type 2 diabetes, patients allocated to 5 years of epalrestat therapy had no worsening of albuminuria and their kidney function decreased at a slower rate when compared to controls [101]. In patients with insulin dependent diabetes, tolrestat decreased eGFR, filtration fraction, and urinary albumin excretion rate when compared to controls, a finding thought to counteract the early changes found in DKD [102]. Tolrestat was later removed from the worldwide market due to its association with hepatic necrosis [103].

Very few hexosamine pathway inhibitors have been studied in DKD. Azaserine is an inhibitor of the rate-limiting enzyme, GFAT. In* in vitro* studies, azaserine decreased VCAM-1 and ICAM-1 expression in hyperglycemic states and enhanced expression of the antioxidant manganese superoxide dismutase levels [104]. This study demonstrated that hyperglycemia independently impaired endothelial cell function via oxidative stress and not solely via the hexosamine pathway; it also demonstrated that azaserine was capable of decreasing GFAT activity but more importantly had antioxidant effects. Rhein is an anthraquinone derived from rhubarb known to decrease hexosamine pathway activity [105]. In a rat mesangial cell line that replicates a diabetic state, rhein decreased TGF-*β*1 and p21 expression and contributed to decreased cellular hypertrophy and ECM synthesis [106]. Benfotiamine is a synthetic thiamine that converts fructose-6-phosphate, AGE precursors, and PKC precursors into pentose-5-phosphate and thus diverts activity from the hexosamine pathway and decreases AGE production and PCK activation [107, 108]. Diabetic rats treated with benfotiamine had suppressed AGE accumulation and decreased vascular endothelial dysfunction and attenuation of nephropathy [109, 110]. However, in a double-blind, randomized, placebo-controlled clinical trial evaluating the effect of benfotiamine on patients with type 2 diabetes and microalbuminuria despite ACEI or ARB therapy, there was no significant difference in urinary albumin excretion between benfotiamine and the placebo-control group [111].

AGE inhibitors were initially encouraging but newer compounds have not been pursued in recent years [37]. The prototype AGE inhibitor, aminoguanidine, reacts with AGE precursors and prevents their formation [112]. It was initially promising in diabetic rat models which demonstrated that aminoguanidine reduced the rise in albuminuria and prevented mesangial expansion when compared to diabetic controls [113]. ACTION I was a randomized, double-blinded, placebo-controlled study in patients with type 1 diabetes with nephropathy. In patients randomized to aminoguanidine, there was a reduction in 24-hour total urine protein (*P* ≤ 0.001) and a trend toward a slower decline in eGFR (*P* = 0.05) [114]. ACTION II was a randomized, double-blind, placebo-controlled trial in patients with type 2 diabetes with renal disease; however it was terminated early due to safety concerns and apparent lack of efficacy [115, 116]. Pyridoxamine is derived from the vitamin B family and inhibits AGE formation and prevents AGE-dependent oxidative damage [18, 117]. In phase II trials of patients with type 1 and type 2 diabetes with overt nephropathy, pyridoxamine significantly reduced the change from baseline in serum creatinine (*P* < 0.03) [118]. Although these findings were not replicated in a randomized, double-blinded, placebo-controlled trial of 317 patients with type 2 diabetes with proteinuric nephropathy, it did find that patients with less renal impairment at baseline experienced a trend toward lower change in serum creatinine from baseline when randomized to pyridoxamine (*P* = 0.05) [119]. Additionally, PIONEER is a phase 3 randomized, double-blind, placebo-controlled, multicenter study currently recruiting patients with type 2 diabetes with nephropathy (defined as urine protein ration > 1200 mg/g) that will compare the time to ≥50% increase in serum creatinine from baseline or time to ESRD in patients randomized to pyridoxamine versus placebo [120].

Other therapeutics have targeted the downstream products of AGEs such as ROS, TGF-B1, and CTGF. Bardoxolone is a potent activator of the nuclear factor erythroid-derived factor 2-related factor 2 (Nrf2) pathway which is a cellular regulator against oxidative species [121]. Bardoxolone was initially evaluated in the BEAM study, a phase 2 randomized, double-blind, placebo-controlled trial in patients with type 2 diabetes with CKD. It demonstrated that bardoxolone significantly improved eGFR in a dose-dependent manner when compared to placebo [122]. The BEACON study was a larger phase 3 study with 2,185 patients with type 2 diabetes and stage 4 CKD which confirmed that bardoxolone improved eGFR, blood pressure, and albuminuria. However it did not reduce progression to ESRD, and unfortunately, bardoxolone also caused an increase in the rate of cardiovascular events and the study was terminated prematurely [123].

Sevelamer is thought to reduce oxidative stress by binding AGEs in the gastrointestinal tract. In a single-center, randomized study which compared administration of calcium carbonate to sevelamer in patients with diabetes with stage 2-4 CKD, sevelamer significantly decreased markers of inflammation and oxidative stress (TNF-*α*, FGF-23, and methylglyoxal levels) and increased antioxidant markers. There was no significant change in eGFR or proteinuria [124]. While the authors concluded that sevelamer may one day be used as an early therapeutic in DKD, this remains to be validated in larger trials.

Sulodexide is a glycosaminoglycan which is thought to inhibit ROS production and TGF-*β*1 expression [18]. It also is an inhibitor of the heparanase enzyme, an enzyme responsible for cleaving heparan sulfate, the main polysaccharide of the GBM [125, 126]. A decreased amount of heparan sulfate in the GBM is thought to alter its selectivity and allow negatively charged macromolecules such as albumin to pass into the urinary space [127]. Overexpression of heparanase in diabetic mouse models increased TNF-*α* expression in kidney tissue and activated kidney-damaging macrophages, whereas heparanase knockout mice with diabetes demonstrated reduced albuminuria [126, 128]. Sulodexide first showed promise in the dose-range finding DiNAS trial which demonstrated that it reduced micro- and macroalbuminuria in patients with both type 1 and 2 diabetes [129]. The Sun-MICRO study was a multicenter placebo-controlled double-blinded study performed in patients with type 2 diabetes with microalbuminuria that failed to show that sulodexide decreased albuminuria [130]. When these results became available, the Sun-MACRO study which was evaluating the effect of sulodexide on patients with type 2 diabetes with overt proteinuria (>900 mg/d) was terminated early. At the time of its termination, 1,029 person-years of follow-up had not detected a significant difference between sulodexide and placebo in progression to ESRD or change in creatinine from baseline [131].

Nicorandil is a known antianginal agent that dilates vessels by opening the ATP-dependent K channel and donating nitric oxide. This same ATP-dependent K channel is expressed in podocytes. Nicorandil is thought to prevent reduced levels of manganese superoxide dismutase and sirtuin-3 (Sirt3), a regulator of the mitochondrial adaptive response to stress, in injured kidneys [132, 133]. In diabetic mouse models, nicorandil was shown to significantly reduce proteinuria, pathologic features of glomerular injury, and protected against podocyte loss [134]. There are no studies to date of nicorandil use in patients with diabetes.

Cilostazol is a phosphodiesterase III inhibitor used to relieve symptoms of claudication in patients with peripheral vascular disease. It has also been shown to decrease ROS* in situ* [135]. In diabetic rats, cilostazol not only significantly decreased ROS, but also significantly decreased albuminuria, glomerular size, and expression of TGF-*β* and NF-*κ*B [136]. In patients with type 2 diabetes randomized to cilostazol, there was a significant decrease in albuminuria as well as a decreased expression of inflammatory markers and adhesion molecules [137, 138] suggesting that cilostazol likely has several favorable effects in the diabetic kidney which remain to be delineated.

Pirfenidone is another TGF-*β* inhibitor that first showed promise in a diabetic murine model. In diabetic mice randomized to pirfenidone, there was a reduction in TGF-*β* production and mesangial matrix expansion, although no change in albuminuria was noted [139]. In a randomized, double-blind, placebo-controlled study in 77 patients with diabetic nephropathy, patients that received low-dose pirfenidone had significantly improved eGFR and decreased markers of fibrosis (TNF, soluble TNF receptor 1, and fibroblast growth factor-23) when compared to placebo whereas no difference was noted in the high-dose group or in albuminuria [140]. The authors suggested that the improvement in eGFR may have been due to a reduction in fibrosis and that antifibrotics may be able to halt and potentially reverse a degree of renal injury.

Tranilast is another antifibrotic agent thought to interfere with the effects of TGF-*β*. It was shown to decrease albuminuria and urinary type IV collagen excretion in patients with diabetes with albuminuria [141]. An analog of tranilast (FT061) is currently undergoing phase I enrollment in Australia. There is also an ongoing phase 2 study evaluating the effects of an anti-TGF-*β* antibody (LY2382770) on the change in serum creatinine levels from baseline to 12 months in patients with type 1 and type 2 diabetes with diabetic nephropathy, the results of which have not yet been reported [142].

FG-3019 is an anti-CTGF monoclonal antibody which demonstrated a non-dose-dependent reduction in albuminuria in phase 1 trails in patients with diabetes with microalbuminuria [143]. Phase 2 trials were halted due to suboptimal study design [144]; however a second phase 1 study of FG-3019 in patients with diabetic nephropathy on background ACEI or ARB has been completed although the results have not yet been reported [142, 145].

Ruboxistaurin is a selective inhibitor of the PKC-*β* isoform and was also promising in diabetic rat models demonstrating improvement in eGFR, albumin excretion rate, and mesangial expansion rate when compared to controls [146, 147]. In a phase 2, randomized, double-blind, placebo-controlled study of ruboxistaurin in patients with type 2 diabetes and persistent albuminuria, patients randomized to ruboxistaurin had a significant decrease in albuminuria while maintaining a stable eGFR and urinary TGF-*β* level, whereas those randomized to placebo had an increase in their albuminuria and urinary TGF-*β* levels as well as a decrease in their eGFR [148, 149]. In larger and longer term studies looking at ruboxistaurin in diabetic retinopathy, the agent was confirmed to have a good safety profile; unfortunately, baseline albuminuria was not measured in this population so it is not possible to know how many patients in this study started out with DKD. Although these early studies were promising enough to support a phase 3 trial of ruboxistaurin evaluating clinical endpoints as mortality, ESRD, and DKD progression, this was halted for business considerations and further development has been postponed [19, 37, 150].

## 10. Therapeutics Targeting the Inflammatory Pathway

As noted above, NF-*κ*B expression in the kidney is associated with inflammation and cell death and leads to interstitial cell infiltration and proteinuria. In diabetic rat models, the thiazolidinedione, pioglitazone, was shown to decrease expression of TGF-*β*, type IV collagen, and ICAM-1, the infiltration of macrophages in kidneys, and albuminuria and glomerular hypertrophy [65]. A recent study looked at the effect of another thiazolidinedione, rosiglitazone, in 28 patients with type 2 diabetes with overt nephropathy. Patients randomized to rosiglitazone had a significant reduction in proteinuria although there was no change in eGFR [151]. There is currently an ongoing phase 4 study evaluating the long-term effects of thiazolidinediones in patients with type 2 diabetes with microalbuminuria to see if the onset of overt nephropathy is significantly delayed when compared to controls [152].

1,25-Dihydroxyvitamin D3 has been shown to block hyperglycemia-induced renal injury by inhibiting NF-*κ*B activation* in vitro* [153]. In humans, there have also been various small studies showing that vitamin D3 has an antiproteinuric effect in patients with diabetes [154–156]. It remains to be seen whether the antiproteinuric effect is due primarily to NF-*κ*B inhibition or a combination of effects from vitamin D.

The JAK/STAT pathway was shown to be inhibited by suppressors of cytokine signaling (SOCS) proteins. Increased expression of SOCS1 and SOCS3 was seen in biopsies of patients with DKD when compared to those with minimal change disease [157]. When SOCS1 and SOC3 adenovirus were delivered into the kidneys of diabetic rats, their renal function significantly improved; they had decreased mesangial expansion, fibrosis, and influx of macrophages, as well as decreased expression of inflammatory and profibrotic proteins [157]. It is unclear if the JAK/STAT pathway will ever be a reasonable target for therapeutics in DKD given its ubiquitous presence in the body and the potential for adverse effects [61]. However, JAK/STAT pathway inhibitors have a long history of safety and efficacy in autoimmune diseases such as rheumatoid arthritis. Baricitinib (LY3009104) is a JAK1/JAK2 inhibitor that initially developed to treat rheumatoid arthritis and is now also being evaluated in a phase 2 study for patients with DKD [158].

The inflammatory cytokines also have far-reaching effects in the body which unfortunately limit their therapeutic targeting. For example, mycophenolate mofetil (MMF) is an immunosuppressant which decreased albuminuria, glomerular macrophage and lymphocyte infiltration, and glomerulosclerosis in diabetic rats [159, 160]. Etanercept and infliximab both inhibit TNF-*α* and also decreased urinary excretion of both albumin and TNF-*α* in diabetic rat models [161, 162]. However, at this time, the use of potent agents such as MMF, etanercept, or infliximab, which carry their own potentially lethal side effects, seems unethical in the treatment of a progressive disease such as DKD.

Like cilostazol, pentoxifylline is a phosphodiesterase inhibitor which also has anti-inflammatory properties. In animal models it decreased expression of TNF-*α*, IL1, IL-6, and interferon-*λ* [61, 69]. In diabetic rats treated with pentoxifylline, there was decreased GBM thickening, podocyte flattening, loss of fenestration in the endothelial cell layer, and albuminuria when compared to controls [69, 163]. Various studies in patients with type 1 and type 2 diabetes have demonstrated the antiproteinuric effects of pentoxifylline [164–166]. A meta-analysis determined that the decrease in proteinuria with pentoxifylline was similar to that of captopril [167, 168] and that the combination of pentoxifylline with an ACEI or ARB had significantly additive antiproteinuric effects [169, 170]. Despite the promising results of pentoxifylline, no large-scale randomized clinical trials have been performed to date that analyze relevant clinical endpoints such as mortality or progression to DKD or ESRD [19]. Until data is available to support the use of pentoxifylline alone or combined with an ACEI or ARB, it is unlikely to become a mainstream therapeutic for DKD.

**Figure 3:**
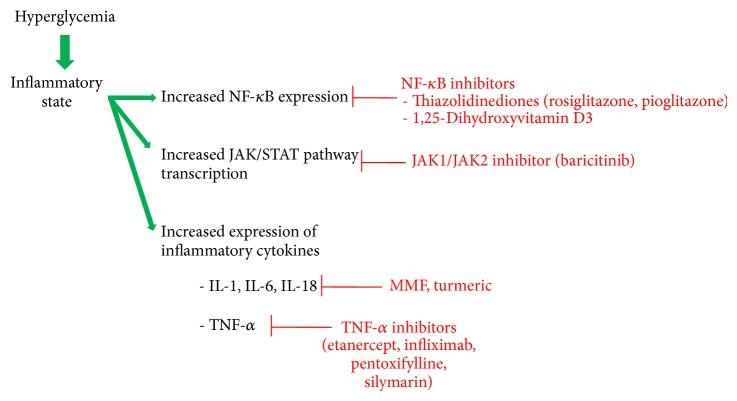
A schematic summary of the therapeutics that have been identified in the inflammatory pathway. The inflammatory state occurs as a result of hyperglycemia and is seen in patients with both type 1 and 2 diabetes.

Interestingly, various complementary therapeutics have been pursued in the quest for improving DKD, including milk thistle, turmeric, and green tea. Silymarin is the main active component found in seeds of milk thistle and has been used since ancient times for a variety of ailments; it is thought to have powerful anti-inflammatory, antioxidant, and antifibrotic properties [171]. In a randomized, double-blind, placebo-controlled trial of 60 patients with type 2 diabetes with macroalbuminuria, silymarin was found to significantly reduce albuminuria and urinary and serum levels of TNF-*α* and malondialdehyde, the latter being a marker of oxidative stress, when compared to controls [171]. Turmeric is a popular South Asian spice of the ginger family and has been shown in experimental models to reduce expression of both TGF-*β* and IL-8 [172, 173]. In a randomized, double-blind, placebo-controlled trial of 40 patients with type 2 diabetes with overt nephropathy, those randomized to curcumin, the active ingredient in turmeric, were noted to have significantly decreased proteinuria, serum levels of TGF-*β*, and serum and urinary levels of IL-8 when compared to controls [174]. Additionally, the antioxidant derived from green tea, epigallocatechin gallate, is currently recruiting patients for a clinical trial to evaluate its effect on albuminuria and oxidative stress in patients with diabetic nephropathy [18, 175]. The therapeutic agents involved in the inflammatory pathway are delineated in Figure 3.

## 11. Therapeutics Targeting the Alternative Pathway

The loss of autophagy appears to be mendable in experimental models. As mentioned above, dietary restriction in diabetic rats improved albuminuria, mesangial expansion, renal fibrosis, expression of TGF-*β*1, fibronectin, collagen type IV, ICAM-1, NF-*κ*B, and Sirt1, the latter being a positive regulator of autophagy [89]. In this particular study, the diabetic rat models were given a 40% restriction of food consumption which lasted 24 weeks. Although such a study in humans has not been reproduced on a large-scale, a recent small study suggested that caloric restriction does play a role in ameliorating DKD. Six obese patients with advanced diabetic nephropathy who were already on an ACEI or ARB were assigned to a 12-week very low calorie ketogenic weight reduction diet and were encouraged to exercise. The patients had a 12% reduction in weight and a significant improvement in serum creatinine and eGFR. Although patients had a non-statistically significant reduction in albuminuria, the authors argue that the trend toward improvement is notable given that the patients were already on a renin-aldosterone axis inhibitor [176]. Although recommending a severe dietary restriction to patients with diabetes may not be a reasonable solution in today's hyperphagic society, targeting autophagy remains a viable option [177]. Sirt1 activators such as resveratrol have been studied in diabetic rat models and were shown to improve proteinuria and renal dysfunction and decrease ROS when compared to controls [178–180]. Whether resveratrol and other activators of Sirt1 will play a role in DKD remains to be seen.

**Figure 4:**
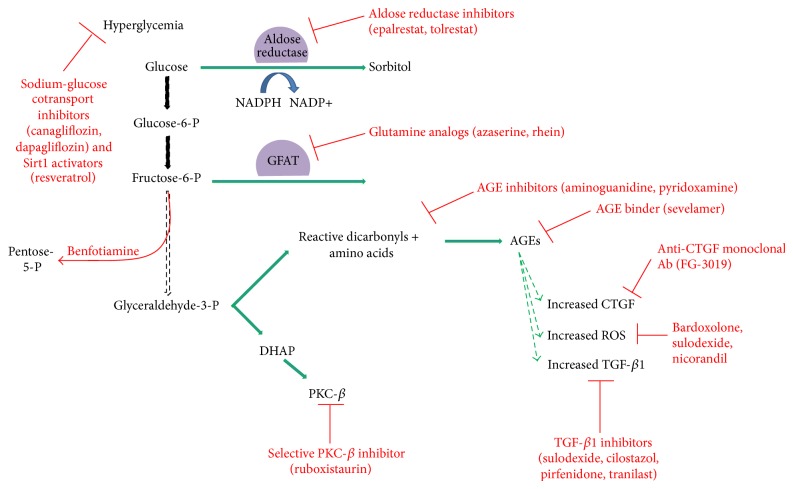
A schematic summary of the various therapeutic agents that have been identified in the metabolic and alternative pathways is shown below. As noted in Figure 2(b), a hyperglycemic milieu shunts glucose metabolism away from the classic glycolysis pathway as is typically seen in patients with type 1 and type 2 diabetes. Sodium-glucose cotransport (SLGT2) inhibitors and Sirt1 activators play a role in alternative pathway and are noted in the upper left. SLGT2 inhibitors combat hyperglycemia by inducing glucosuria, whereas Sirt1 activators are thought to upregulate the highly conserved mechanism, autophagy.

Finally, sodium-glucose cotransporter 2 (SGLT2) inhibitors are of potential therapeutic benefit in DKD. They inhibit the reabsorption of glucose in the proximal tubule, reduce HbA1c levels by 0.5–1% [90], and contribute to weight loss as a result of glucosuria and improve systolic and diastolic blood pressures as a result of osmotic diuresis [181, 182]. In diabetic mouse models, dapagliflozin, a SGLT2 inhibitor, reduced hyperglycemia, albuminuria, the expression of inflammatory cytokines and oxidative stress, glomerular mesangial expansion, and interstitial fibrosis when compared to controls. Currently, two gliflozins, canagliflozin and dapagliflozin, have been approved by the FDA for use in the treatment of type 2 diabetes. Unfortunately, the gliflozin trials understandably focus on diabetic markers of disease and there is little information on the benefits that these medications may exert on the kidney. To date, there have been five randomized, double-blind, placebo-controlled trials that have evaluated the effect of gliflozins on eGFR and the results are mixed. Of three trials on dapagliflozin, eGFR was unchanged in a 12-week study when compared to controls [183] whereas in a second study serum creatinine, creatinine clearance, and eGFR decreased over a 48-week period when compared to controls [184]. In the third study, eGFR decreased over the first week but then stabilized and at the end of the 104-week follow-up, there was no significant difference in serum creatinine or eGFR when compared to controls. Additionally, albuminuria and proteinuria were slightly improved in the dapagliflozin group when compared to controls [185]. The first canagliflozin trial demonstrated a reduction in eGFR after 52 weeks compared to baseline; however the reduction was less than that in the glimepiride control group [186]. The second canagliflozin trial also noted a reduction in eGFR after 52 weeks when compared to placebo, but this was only significant in the high dose canagliflozin arm. Additionally, there was a trend toward improvement in albuminuria, although this was again only seen in the high dose canagliflozin arm [187]. At this time, it remains unknown what effect, if any, gliflozins have on mitigating DKD. It is possible that the reduction in eGFR that is seen early on with their treatment is a result of osmotic diuresis and the consequent intravascular volume depletion that they cause. It will be possible that future long-term studies may demonstrate a similarity to ACEIs and ARBs in that they cause an initial decline in eGFR but have long-term renoprotective effects. The therapeutic agents involved in the alternative pathway are shown in Figure 4.

## 12. Commentary

Despite the tremendous advancement in delineating the pathways that contribute to DKD, clinicians are still a long way away from having a new drug in their prescribing arsenal. Many of the above therapeutics have been successful in experimental models, yet few have proven sufficiently efficacious to be brought into the mainstream management of DKD. Perhaps one of the reasons that finding a new drug for DKD has proven difficult is that there still is not a perfect marker for DKD. While albuminuria is considered the gold standard for diagnosing DKD and remains the strongest predictor of mortality in diabetes, it does not detect almost half of patients with diabetes that progress to DKD whilst remaining normoalbuminuric [23, 188]. Perhaps the prolific research in tubular biomarkers will reveal one that is more specific for DKD than albuminuria. It would be more useful yet to find a marker of DKD that precedes the histopathologic damage that has already occurred once albuminuria is noted. A study by Lurbe et al. demonstrated that an increase in systolic blood pressure during sleep preceded the development of microalbuminuria in patients with type 1 diabetes [189]. It would be remarkable to find that an inexpensive and noninvasive maneuver such as measuring blood pressure during sleep could identify future cases of DKD before they develop. It would certainly be less expensive and noninvasive than performing more kidney biopsies in patients with diabetes. However, performing kidney biopsy in this setting may also be beneficial to patients and clinicians alike. In a retrospective study of 620 patients with type 1 and type 2 diabetes that had undergone biopsy, only 37% had isolated DKD. Thirty-six percent of patients had nondiabetic renal disease, which included glomerulonephritides, acute tubular necrosis, and hypertensive nephrosclerosis; the remaining 27% had nondiabetic renal disease superimposed on DKD [18, 190]. Until clinicians are better at discerning the cause of kidney disease in their patients, obtaining a histopathologic diagnosis may prove fruitful as it would allow them to identify those patients with or without DKD and guide their care accordingly.

Ultimately, the treatment of DKD will likely require a multifaceted approach given the numerous pathways involved in the diabetic kidney. Perhaps one day, “triple therapy” will also refer to the multipronged approach necessary to tackle DKD.
